# Prevalence and risk factors for resistant hypertension among hypertensive patients from a developing country

**DOI:** 10.1186/1756-0500-6-373

**Published:** 2013-09-21

**Authors:** WA Nuwan Kumara, Thisara Perera, Mekhala Dissanayake, Priyanga Ranasinghe, Godwin R Constantine

**Affiliations:** 1 Department of Clinical Medicine, Faculty of Medicine, University of Colombo, Colombo, Sri Lanka; 2 Ministry of Health Care and Nutrition, Colombo, Sri Lanka; 3 Department of Pharmacology, Faculty of Medicine, University of Colombo, Colombo, Sri Lanka

**Keywords:** Resistant hypertension, Prevalence, Risk factors, Sri Lanka, South Asia, Developing country

## Abstract

**Background:**

To study the prevalence and define deferential risk factors for ‘Resistant’ hypertension (RHT) in a hypertensive population of South Asian origin.

**Methods:**

A descriptive cross-sectional study was carried out among hypertensive patients attending clinics at the Cardiology Unit, Colombo from July-October 2009. All the patients with hypertension who provided informed written consent were recruited to the study (n = 277). A pre-tested interviewer-administered questionnaire was used for data collection. A binary logistic-regression analysis was performed in all patients with ‘presence of RHT’ as the dichotomous dependent variable and other independent co-variants.

**Results:**

Mean age was 61 ± 10.3 years and 50.2% were males. The mean of average systolic and diastolic blood pressures (BP) were 133.04 ± 12.91 mmHg and 81.07 ± 6.41 mmHg respectively. Uncontrolled BP was present in 41.1% (n = 114) of patients, of which RHT was present in 19.1% (n = 53). Uncontrolled BP were due to ‘therapeutic inertia’ in 27.8% of the study population. Those with diabetes mellitus, obesity (BMI > 27.5 kg/m^2^) and those who were older than 55 years were significantly higher in the RHT group than in the non-RHT group. In the binary logistic regression analysis older age (OR:1.36), longer duration of hypertension (OR:1.76), presence of diabetes mellitus (OR:1.67) and being obese (OR:1.84) were significantly associated with RHT.

**Conclusion:**

A significant proportion of the hypertensive patients were having uncontrolled hypertension. Nearly 1/5^th^ of the population was suffering from RHT, which was significantly associated with the presence of obesity and diabetes mellitus. Therapeutic inertia seems to contribute significantly towards the presence of uncontrolled BP.

## Background

Hypertension is a common non-communicable disease that is prevalent worldwide; it leads to numerous disabling complications such as stroke, atherosclerosis, retinopathy, chronic kidney disease and cardiac failure [1]. Majority of patients (>90%) with hypertension suffer from essential or primary hypertension, while the remaining minority have secondary hypertension. Long term optimization and control of blood pressure is essential to avoid morbidity and mortality in these patients. However it is not uncommon to see poorly controlled hypertension and it is estimated that only 1/3 of patients on treatment have their blood pressures well controlled [2]. Most causes for poor control is well known, however a considerable percentage falls into a category known as ‘resistant hypertension’ of which pathophysiology and risk factors are not fully understood [3]. Resistant hypertension is defined as "Suboptimal control of blood pressure despite using three antihypertensive agents inclusive of a diuretic, and patients who need 4 or more drugs to control blood pressure" [3]. Despite having guidelines on management of resistant hypertension, it has become a problem to control blood pressure up to recommended levels, possibly due to poor understanding of pathophysiology and risk factors.

Studies have shown that older age, obesity, excessive use of alcohol, and high sodium intake are strongly correlated with poor control of hypertension [4,5]. Patient factors such as compliance and knowledge, and health care system factors like limitation of resources and lack of reminders of appointments also plays a major role in poor blood pressure control [6-10]. Managing resistant hypertension is difficult and involves expensive testing to look for underlying secondary causes. Furthermore, patients with uncontrolled blood pressure are more likely to have target organ damage and have higher cardiovascular risks than patients with well controlled blood pressure [11]. Uncontrolled blood pressure affects patients mental, physical and social well being, while also increasing the health care expenditure of a country.

Sri Lanka is a middle income developing country in the South Asian region with a population of over 20 million. In 2005, nearly 1/5^th^ of the population of Sri Lanka was suffering from hypertension and the prevalence is expected to increase further in the coming decades [12]. Furthermore, cardio- and cerebro-vascular diseases for which hypertension is an important risk factor, are the leading causes of hospital deaths in Sri Lanka, and cause specific mortality rates are higher among Sri Lankans in comparison to affluent countries [13]. The prevalence rate for hypertension in urban India is 29-45% in men and 25-38% in women, while data from other South Asian countries are sparse [14]. Furthermore, studies from developing countries have shown that hypertension is more common among South Asian immigrants than among the native white population [15]. Genetic and environmental risk factors are important in aetio-pathogenesis of hypertension and genetic variations could be the reason for these differences in prevalence. Indeed, approximately a quarter of blood pressure associated loci reported appear to be common in people of South Asian ethnicity [16].

There are numerous studies and published guidelines from developed countries on the prevalence, risk factors and management of ‘Resistant’ hypertension. However, presently there are no studies from developing South Asian countries. The present study aims to study the prevalence of ‘Resistant’ hypertension in a hypertensive population of South Asian origin and define deferential risk factors in the same population.

## Methods

This descriptive cross sectional study was conducted over a period of 3 months from 1st of March 2009 to 30th of May 2009. Ethical approval for the study was obtained from the Ethics Review Committee, Faculty of Medicine, University of Colombo, Sri Lanka.

### Study population and sampling

Patients were recruited from a cohort of hypertensive patients attending follow-up clinics at the Institute of Cardiology of the National Hospital of Sri Lanka (NHSL). Situated in Colombo the NHSL is the largest tertiary care hospital in Sri Lanka. There were about 50 hypertensive patients visiting daily (excluding Sundays) for follow up to the cardiology clinic, these patients are given clinic numbers on the first come basis and they come to clinics usually once per month. In order to choose the sample for our study we used a random sampling technique. In each week we selected three random days to visit the clinic. On each day of study we first selected all the patients with hypertension from the daily clinic register. These patients were then arranged in ascending order according to their clinic number. The first patient was chosen randomly from hypertensive patients 1 to 10 and then every third patient thereafter was invited for the study. Patients who gave the informed written consent were included in the study. The patients who visited the clinic for the 1^st^ time, patients who were too ill/unable to answer the questionnaire and patients who did not consent were excluded from the study.

### Study instrument and data collection

A pre-tested expert-validated interviewer administered questionnaire was used for data collection (Annexure 1). The following data were collected; socio-demographic details, duration of disease, medication history, risk factors, complications and other co morbidities. The following risk factors were evaluated; history of smoking, alcohol consumption, drugs (Non-Steroidal Anti-Inflammatory Drugs, Steroids and Oral Contraceptive Pills), family history, high salt intake and presence of obesity. The antihypertensive drugs currently used by the patients were recorded according to their classes and drugs used for other co-morbidities were also documented. Patients’ compliance to treatment was also evaluated.

Height was measured as the maximum distance to the uppermost position on the head from heels, with the individual standing barefoot and in full inspiration using Harpenden stadiometers (Chasmors Ltd, London, UK) to the nearest 0.1 cm. Body weight was measured using a SALTER 920 digital weighing scale (SALTER Ltd, Tonbridge, UK) to the nearest 0.1 kg. Body Mass Index (BMI) was calculated as weight in kilograms divided by height squared in meters (kg.m^-2^). Two consecutive resting seated blood pressures were recorded 10 minutes apart, manually using mercury sphygmomanometers. Patients’ previous 3 blood pressure values were taken from their medical records in clinic books.

### Definitions

Resistant hypertension was defined as “Suboptimal blood pressure despite using three antihypertensive agents inclusive of a diuretic, and patients who need 4 or more drugs to control blood pressure also called resistant hypertension” [3]. Hypertension treatment targets were < 140/90 mmHg for patients without any co-morbidities and < 130/90 mmHg for patients with diabetes mellitus and renal disease [17]. Obesity was defined as BMI ≥ 27.5 kg/m^2^, based on WHO criteria for Asians [18]. High salt intake was defined as an intake of sodium > 3 mg/day based on Food Frequency Questionnaires. Current cigarette smokers were defined as adults aged ≥18 years who reported having smoked ≥100 cigarettes during their lifetime and who now smoke every day or some days [19]. Current alcohol consumption was defined as ≥ 1 alcoholic drink per month [20]. Presence of diabetes mellitus, ischaemic heart disease, chronic kidney disease and hyperlipidaemia were confirmed by perusal of previous clinic records of the patients.

### Data analysis

Data were analyzed using SPSS version 15 statistical software package (SPSS Inc., Chicago, IL, USA). The significance of the differences between means was tested using z-test. In all analyses a p values < 0.05 was considered statistically significant. A binary logistic regression analysis was performed in all patients with ‘presence of Resistant hypertension (RHT)’ as the dichotomous dependent variable (0 = RHT absent; 1 = RHT present) and age, gender (0 = female, 1 = male), duration of hypertension, current cigarette smoking (0 = no, 1 = yes), current alcohol consumption (0 = no, 1 = yes), high salt intake (0 = no, 1 = yes), diabetes mellitus (0 = absent, 1 = present), ischaemic heart disease (0 = absent, 1 = present), hyperlipidaemia (0 = absent, 1 = present), chronic kidney disease (0 = absent, 1 = present) and obesity (0 = BMI < 27.5, 1 = BMI ≥ 27.5) as the independent variables (co-variants).

## Results

### Socio-demographic characteristics

Three hundred and ten adults with hypertension were invited for the study, of which 277 consented to participate in the study and completed the questionnaires (response rate – 86.6%). Mean age was 61 ± 10.3 years (range 25–83), and 50.2% were males. Majority of the study population (75.5%) were the age of 55 years. Mean duration of hypertension was 9.2 years (range 1–38), majority of the patients were having hypertension for ≤ 9 years (n = 152/54.9%). The mean of average systolic and diastolic blood pressures of the population was 133.04 ± 12.91 mmHg and 81.07 ± 6.41 mmHg respectively and 73.3% (n = 203) of them were admitted to hospital at least once due to a complication arising from hypertension (heart failure, cardio-/cerebro-vascular disease, renal failure, hypertensive emergency, and etc.). The mean BMI in the study population was 25.02 ± 4.52 kg/m^2^. Majority of the study population (n = 245/88.4%) had one or more co morbidities and ischemic heart disease (n = 214/77.3%), hyperlipidaemia (n = 144/52.0%) and diabetes mellitus (n = 118/42.6%) were the commonest co-morbidities (Table 1).

**Table 1:** Co-morbidities in all adults, in those with and without resistant hypertension

**Co-morbid disease**	**Number of patients with hypertension (%)**			p value*
	**All**	**Resistant**	**Non resistant**	
	**(n = 277)**	**(n = 53)**	**(n = 224)**	
DM	118 (42.6%)	37 (69.9%)	81 (36.1%)	< 0.001
IHD	214 (77.3%)	48 (90.6%)	161 (71.9%)	< 0.01
Hyperlipideamia	144 (52%)	26 (49.1%)	118 (52.7%)	NS
CKD	9 (3.2%)	3 (5.7%)	6 (2.7%)	NS

*CKD* Chronic Kidney Disease, *DM* Diabetes Mellitus, *IHD* Ischaemic Heart Disease, *NS* Not Significant; *, p values for ‘Resistant’ Vs. ‘Non-resistant’ groups.

### Prevalence of resistant hypertension

Either systolic (≥140 mmHg or >130 mmHg in diabetics) or diastolic (≥90 mmHg or >80 mmHg in diabetics) blood pressure values measured during two recent clinic visits one month apart, were high in both visit in 41.1% (n = 114) of patients. Among these 114 patients, 37 (32.4%) of them were using 3 antihypertensive drugs including a diuretic, a proportion of 13.3% when considering the entire study population. Another 5.7% (n = 16) of patients who were having normal blood pressures, were using 4 or more anti-hypertensive drugs. Hence the percentage of patients with resistant hypertension according to the definition was 19.1% (n = 53) of the entire study population. The phenomenon of doctors’ failing to intensify medication regimens at encounters with patients, who have an uncontrolled risk factor, has been described recently as ‘therapeutic inertia’ [21]. Accordingly the remaining 77 of the 114 patients with uncontrolled blood pressures were without optimum anti-hypertensive therapy. Hence, the prevalence of therapeutic inertia in our study sample is 27.8%.

### Anti-hypertensive therapy

All the patients with resistant hypertension were compliant with their therapeutic regime and majority (93.9%) of the patients in the non-resistant hypertensive group was also compliant. Table 2 summarizes the anti-hypertensive drugs and other drugs used in the study population. Accordingly, the most commonly used drug in the population was anti-platelets (72.6%). The most commonly used anti-hypertensive drug was ACE inhibitors (54.5%) followed by β-blockers (51.6%) and Calcium Channel Blockers (CCBs) (47.3%). In the resistant hypertension group, the most commonly used anti-hypertensive drug was β-blockers (71.7%) followed by ACE inhibitors (69.8%) and CCBs (54.7%). The usage of ACE inhibitors, α-blockers, β-blockers, furosemide, spiranolactone and thiazide diuretics were significantly more in the resistant hypertension group than in the non-resistant hypertension group (Table 2).

**Table 2:** Usage of drugs in those with and without resistant hypertension

**Name of drug**	**Number of patients (%)**			p value*
	**All**	**Resistant**	**Non resistant**	
	**(n = 277)**	**(n = 53)**	**(n = 224)**	
Anti-hypertensives				
ACEI	151 (54.5%)	37 (69.8%)	114 (50.9%)	<0.05
α-blockers	21 (7.6%)	14 (26.4%)	7 (3.1%)	<0.001
ARB	94 (33.9%)	22 (41.5%)	72 (32.1%)	NS
β-blockers	143 (51.6%)	38 (71.7%)	105 (46.9%)	<0.01
CCBs	131 (47.3%)	29 (54.7%)	102 (45.5%)	NS
Furosemide	59 (21.3%)	26 (49.1%)	33 (14.7%)	<0.001
Spiranolactone	8 (2.9%)	6 (11.3%)	2 (0.9%)	<0.01
Thiazide	34 (12.3%)	16 (30.2%)	18 (8.0%)	<0.001
Other drugs				
Anti platelets	201 (72.6%)	37 (69.8%)	174 (77.7%)	NS
Lipid lowering drugs	152 (54.9%)	25 (47.1%)	127 (56.7%)	NS
Nitrates	139 (50.2%)	26 (49.1%)	113 (50.4%)	NS

*ACEI* Antgiotensin Converting Enzyme Inhibitors, *ARB* Angiotensin-II Receptor Blockers, *CCB* Calcium Channel Blockers, *NS* Not Significant; *, p values for ‘Resistant’ Vs. ‘Non-resistant’ groups.

### Risk factors

There were no significant difference observed between the resistant hypertension and non-resistant hypertension groups for the following modifiable and non-modifiable cardio-vascular risk factors; gender, family history, high salt intake, tobacco smoking, alcohol consumption and hyperlipidaemia (Table 3). Those with diabetes mellitus, obesity (BMI > 27.5 kg/m^2^) and those who were older than 55 years were significantly higher in the resistant hypertension group than in the non-resistant hypertension group (Table 3). In the binary logistic regression analysis older age (OR: 1.36, 95%CI 1.14–1.56), longer duration of hypertension (OR: 1.76, 95% CI 1.26– 2.28), presence of diabetes mellitus (OR: 1.67, 95% CI 1.31–1.97) and being obese (OR: 1.84, 95% CI 1.04–3.26) were significantly associated with resistant hypertension (Table 4).

**Table 3:** Cardiovascular risk factors in those with and without resistant hypertension

**Risk factors**	**Number of patients (%)**			*p value
	**All**	**Resistant**	**Non resistant**	
	**(n = 277)**	**(n = 53)**	**(n = 224)**	
Non modifiable				
Age (> 55 years)	202 (72.9%)	46 (86.8%)	156 (69.6%)	< 0.05
Gender – Male	139 (50.2%)	28 (52.8%)	111 (49.5%)	NS
Gender – Female	138 (49.8%)	25 (47.2%)	113 (50.4%)	NS
Family history	126 (45.5%)	29 (54.7%)	97 (43.3%)	NS
Modifiable				
High salt intake	61 (22.0%)	12 (22.6%)	49 (21.9%)	NS
Tobacco smoking	16 (5.8%)	1 (1.9%)	15 (6.7%)	NS
Hyperlipidaemia	142 (51.3%)	24 (45.3%)	118 (52.7%)	NS
Diabetes mellitus	111 (40.1%)	30 (56.6%)	81 (36.2%)	< 0.01
Obesity (BMI > 27.5 kg/m^2^)	185 (66.8%)	42 (79.2%)	143 (63.8%)	< 0.05
Alcohol consumption	29 (10.5%)	7 (13.2%)	22 (9.8%)	NS

*BMI* Body Mass Index, *NS* Not Significant; *, p values for ‘Resistant’ Vs. ‘Non-resistant’ groups.

**Table 4:** Binary logistic regression analysis of factors associated with resistant hypertension

**Risk factor**	**β-coefficient (95% CI)**	**p value**
Age	1.36 (1.14 – 1.56)	<0.05
Gender – female	Reference	
male	1.52 (0.67 – 3.47)	NS
Duration of hypertension	1.76 (1.26 – 2.28)	<0.05
Current smoking - non-smoker	Reference	
smoker	1.51 (0.25 – 9.01)	NS
Alcohol consumption - no	Reference	
yes	2.81 (0.96 – 8.20)	NS
Diabetes Mellitus – absent	Reference	
present	1.67 (1.31 – 1.97)	<0.05
IHD – absent	Reference	
present	0.59 (0.12 – 2.50)	NS
CKD - absent	Reference	
present	0.79 (0.39 – 1.58)	NS
Hyperlipidaemia - absent	Reference	
present	1.16 (0.54 – 2.48)	NS
Obesity - BMI <27.5	Reference	
BMI > =27.5	1.84 (1.04 – 3.26)	<0.05
High salt intake – absent	Reference	
present	1.96 (0.94 – 2.95)	NS

*BMI* Body Mass Index, *CKD* Choric Kidney Disease, *IHD* Ischaemic Heart Disease.

## Discussion

This is the first report evaluating the control of blood pressure from Sri Lanka, a developing country in the South Asian region. Our results demonstrate that blood pressure control rates are suboptimal in 41.1% of the local hypertensive population, similar to reports from developed western countries [22-24]. This sub optimal hypertension control includes two different entities; uncontrolled/poorly controlled hypertension and resistant hypertension. Uncontrolled hypertension is lack of blood pressure control secondary to poor adherence and/or an inadequate therapeutic regimen, while treatment resistance is suboptimal blood pressure despite using optimal therapy. Many studies have suggested the prevalence of uncontrolled hypertension to be around 50% of patients being treated for hypertension [25,26]. In the present study, deficiencies in the quality of hypertension management were observed despite the fact that patients were assessed frequently and had satisfactory compliance. The proportion of poorly controlled hypertensive patients with sub optimal drug management was 27.8%. It is the physicians’ failure to increase the intensity of treatment among patients with uncontrolled hypertension, a phenomenon known as therapeutic inertia. Distinguishing therapeutic inertia from other causes for uncontrolled hypertension is an important initial step to identify strategies to improve care offered to these patients.

Majority of patients in both resistant (79.2%) and non-resistant (63.8%) hypertension groups were obese. Obesity is recognized as the sixth most important risk factor contributing to the overall burden of disease worldwide [27]. It is said that compared with year 2000, the number of adults with hypertension is predicted to increase by 60% to a total of 1.56 billion by year 2025 [28]. Furthermore, more than 1 billion adults and 10% of children are now classified as either overweight or obese [27]. Studies have shown that the cardiovascular risks in those with obesity are not significantly increased unless hypertension is present [29]. This observation emphasizes the role of hypertension as a mediator through which obesity may cause cardiovascular disease. Our results also demonstrate that obesity was a significant factor associated with resistant hypertension in the logistic regression analysis. Obesity is associated with more severe hypertension, a need for an increased number of medications and a decreased likelihood of achieving blood pressure control [30]. The impact of body weight change on the prognosis in these patients is potentially of relevance when planning future treatment strategies for uncontrolled hypertension and its cardiovascular consequences.

This epidemic of obesity and obesity-related hypertension is paralleled by an alarming increase in the incidence of diabetes mellitus and chronic kidney disease. We observed a statistically significant relationship between diabetes mellitus and resistant hypertension in the logistic regression analysis. Hypertension in diabetics interferes with the rate of development and progression of diabetic complications, which in turn aggravates the hypertensive disease. It appears to be universally accepted that the tight treatment regimes for hypertension in diabetics reduces cardiovascular risk and slows the rate of progression of diabetic complications such as diabetic nephropathy. Hypertension is usually linked with renal disease and it is both a cause and a complication of hypertension. However in our study sample the number of hypertensive’s with renal disease were minimal, probably due to the fact that there are specialized clinic for patients with renal disease at the NHSL, while we conducted the study in a specialized cardiology clinic. Furthermore, blood pressure, plasma glucose, and lipids are continuous variables that exert a dose-dependent effect on cardiovascular risk [31]. Hence it would be of interest to see the results of the present analysis conducted considering these parameters as continuous variables rather than discreet variables in the future.

Resistant hypertension represents a different phenotype to the general population and it is reasonable to assume that genetic factors play a greater role in pathogenesis. In one of the few genetic evaluations of patients with resistant hypertension, investigators in Finland found that 2β ENaC and γENaC gene variants were significantly more prevalent in the patients with resistant hypertension than in the normotensive controls [32]. A particular allele of the CYP3A5 enzyme that plays an important role in the metabolism of cortisol and corticosterone has been associated in African-American patients with higher systolic blood pressure levels in normotensive participants and hypertension more resistant to treatment [33,34]. Inducible nitric oxide synthase (iNOS) is another important enzyme regulating blood pressure, studies have shown that the g.2087G > A polymorphism in the iNOS gene affects the susceptibility to hypertension. Moreover, the S-C-A haplotype is also associated with responsiveness to antihypertensive therapy [35]. The calcium/calmodulin-dependent kinase IV (CaMKIV) seems to be involved in blood pressure regulation mediated via the control of endothelial nitric oxide synthase activity [36]. In addition the Angiotensinogen AGT 235 T allele has also been shown to be an independent risk factor for resistant hypertension [37]. The heptahelical G-protein-coupled receptors (GPCRs) represent one of the largest classes of cell-surface receptors, a wide variety of GPCRs are involved in blood pressure control. In addition several intermediaries involved in the GPCR desensitization process, like the G-protein-coupled receptor kinases (GRKs) are important in the regulation of vascular tone [38]. Of the seven mammalian GRKs, GRK2 seems to be the most relevant isoform at the cardiovascular level [38]. Identification of genetic influences on resistance to current therapies might also lead to development of new therapeutic targets. However it is important to understand that a single genetic variant may not reveal significant associations with resistant hypertension because their effects may be dependent on gene-gene or gene-environment interactions.

There are a wide range of anti-hypertensives available for the treatment of hypertension. Among them diuretics play major role in blood pressure control. However most of the patients (63.5%) in our study sample were not on any diuretic, including furosemide, spiranolactone and thiazide diuretics. It has been said that combinations of the thiazide-type and potassium-sparing subclasses may be highly effective, providing nearly optimal therapy for some, and might be considered more often in the treatment of hypertension [39]. Treatment of hypertension using a diuretic-based strategy has been effective in preventing stroke and cardiac disease, a consistent finding from the earliest randomized clinical trials (1960s) to present-day large multi-centered studies such as ALLHAT [40]. Thirteen randomized controlled trials have shown that first-line β-blockers for elevated blood pressure were not as good at decreasing mortality and morbidity as other classes of drugs such as thiazides, CCBs and ACE inhibitors [41]. In our study sample β-blockers were the second commonly used anti-hypertensive and there were 9 patients on sole β-blockers therapy of which 4 had uncontrolled blood pressure. ACE inhibitors are some of the most commonly prescribed medications for hypertension. ACE inhibitors are seen as more appropriate for first-line use when other high-risk conditions are present, such as diabetes. It is clear that it is an important role in the treatment of hypertension. In our study sample ACE inhibitors were the most commonly used anti-hypertensive drug. There were 13 patients on sole ACE inhibitor therapy, out of which 6 were having diabetes mellitus and 7 had uncontrolled blood pressure.

There are several limitations that need to be kept in mind when drawing conclusions from the present study. The cross-sectional design of our study limits the inference of causality for the risk factors identified. Therefore, it is important to conduct prospective studies on resistant hypertension and look for causality. There was also incomplete documentation on drug-prescribing decisions and regarding the hospital admissions due to disease complications. It may have led to an underestimation of the control of disease and medication intensifications. In addition, lack of diagnostic laboratory tests may have led to under ascertainment of co-morbidities. Furthermore, it is said that the mental stress is important as an aetiological agent for resistant hypertension. However we did not evaluate the patients stress levels in the present study. In addition although risk factors such as Diabetes, Ischaemic Heart Disease and Chronic Kidney Disease were considered their severity was not evaluated [42].

## Conclusions

A significant proportion of the hypertensive patients were identified as having uncontrolled hypertension. Nearly one fifth of the population was suffering from RHT, which was significantly associated with the presence of obesity and diabetes mellitus. Therapeutic inertia seems to contribute significantly towards the presence of uncontrolled blood pressure and its role and causative factors needs further evaluation.

## Competing interest

The authors declare that they have no competing interests.

## Authors’ contributions

WANK, TP, MD, PR and GRC made substantial contribution to conception and study design. TP, MD and WANK were involved in data collection. GRC, PR and TP were involved in refining the study design, statistical analysis and drafting the manuscript. TP and PR critically revised the manuscript. All authors read and approved the final manuscript.
